# Sub-Low Temperature Preconditioning Induced Cold Signaling and Antiviral Defenses Correlate with Reduced TSWV Accumulation in Tomato

**DOI:** 10.3390/plants15132058

**Published:** 2026-07-02

**Authors:** Wei Guo, Shengjun Xu, Zengguo He, Liu Yang, Junhong Chen, Yahan Chen, Xiang Wang, Xi Lei, Pengyue Zhu, Fuxia Dai, Gaoyi Liu, Huan Luo, Chen Zhou, Ning Luo, Huixia Li

**Affiliations:** 1 College of Plant Protection, Gansu Agricultural University, Lanzhou 730070, China; 17384880633@163.com (L.Y.); juniorc1110@163.com (J.C.); yahanch@163.com (Y.C.); 13753113093@163.com (P.Z.); 18893720126@163.com (F.D.); 20231802039@st.gsau.edu.cn (G.L.); 20231802043@st.gsau.edu.cn (H.L.); 13830814280@163.com (C.Z.); 14249@gsau.edu.cn (N.L.); lihx@gsau.edu.cn (H.L.); 2 Institute of Plant Protection, Gansu Academy of Agricultural Sciences, Lanzhou 730070, China; xusj1001@aliyun.com; 3 Gulang County Agricultural Technology Extension Center, Wuwei 733100, China; hezengguo274768916@163.com; 4 Forestry Sci-Tech Extension Station of Gansu Province, Lanzhou 730046, China; 18298368632@163.com (X.W.); leixi0813@163.com (X.L.)

**Keywords:** tomato, sub-low temperature, *Tomato spotted wilt virus* (TSWV), hormone signaling, antiviral defense

## Abstract

Tomato spotted wilt disease, caused by *Tomato spotted wilt virus* (TSWV), poses a severe, widespread threat to commercial crop production. Triggering endogenous plant defense capacity serves as a promising tactic for sustainable disease control. Environmental factors, such as temperature, can modulate plant metabolic activities to suppress disease development. To characterize how sub-low temperatures shape tomato responses to TSWV, we maintained tomato seedlings under three regimens: room temperature (25 °C) and two sub-low temperatures (15 °C and 10 °C). Subsequent viral inoculation and phenotypic monitoring revealed that 15 °C pretreatment was associated with markedly reduced TSWV disease severity. RT-qPCR quantification further demonstrated that this 15 °C priming correlated with diminished accumulation of the viral *NSs* gene. Transcriptome profiling indicated that the 15 °C regime was linked to coordinated activation of cold acclimation pathways, hormone signaling cascades, and antiviral defense programs, which correlated with reduced TSWV accumulation. Specifically, 15 °C conditioning was correlated with the induction of a cold-responsive transcriptional network that overlaps with plant antiviral immune responses. This activated transcriptional signature is putatively associated with the stimulation of defensive cascades, maintenance of cellular homeostasis, and lowered viral accumulation, which collectively align with alleviated spotted wilt symptoms. To our knowledge, this work presents the first correlative evidence associating 15 °C sub-low temperature preconditioning with reduced TSWV accumulation in tomato. Collectively, these observations highlight the potential of temperature manipulation as a chemical-free disease mitigation approach and provide a valuable reference for sustainable crop protection strategies.

## 1. Introduction

Tomato (*Solanum lycopersicum* L.) is one of the most economically important vegetable crops worldwide, serving as a model plant for studying plant stress responses and pathogen interactions due to its short growth cycle, clear genetic background, and abundant genomic resources [1]. In natural environments, tomato plants frequently encounter a combination of biotic stresses and abiotic stresses, which severely restrict plant growth, development, and productivity, and pose substantial challenges to sustainable tomato production [2]. It is predicted that climate change will increase the frequency of extreme weather events, thereby threatening crop production in agriculture [3]. Among biotic constraints, *Tomato spotted wilt virus* (TSWV) is distributed globally. This virus can infect over 1000 plant species across 85 families and is regarded as one of the ten most destructive plant viruses worldwide [4,5,6,7]. Since its first discovery in Guangzhou, China, in 1989, TSWV has spread to major tomato-producing areas across 18 provinces and municipalities, including Yunnan, Shaanxi, Ningxia and Shandong. The highly endemic core regions are primarily located in Yunnan, the Guanzhong Plain of Shaanxi, and Yinchuan in Ningxia, with the virus affecting both open-field and greenhouse cultivation systems. Within greenhouse systems, the incidence rate of summer and autumn crops is the highest, which can reach 30–65%, while the open-field crops are generally affected in the whole growing season from March to October. Notably, TSWV variants that have overcome the *Sw-5* resistance gene have already emerged. At the same time, the fruit commodity rate can decrease by more than 30% [8,9,10,11,12]. Therefore, exploring novel and sustainable strategies to enhance tomato resistance to TSWV is urgently required to ensure global food security and agricultural sustainability.

With the deepening of research on individual stress signal transduction pathways, recent studies have begun to reveal the molecular intersection between biotic and abiotic stress responses, as well as the regulatory principles in complex stress responses [13]. Abiotic stress harms plant growth and development. Factors such as high temperature, low temperature, drought, and salinity can all induce plant diseases, resulting in reduced yields and decreased quality. However, studies have shown that moderate abiotic stress can induce plant resistance, thereby mitigating the occurrence of diseases. For example, hydrogen peroxide (H_2_O_2_), as a signaling molecule, enhances the natural defense mechanism of plants by regulating their overall stress response and metabolism under abiotic stresses such as drought, salinity, and alkalinity [14]. When facing abiotic stresses (such as drought and low phosphorus), plants regulate their immune defense through the ABA signaling pathway and transcription factors (such as *MYC2*), thereby balancing growth and disease resistance in adverse conditions [15]. These studies provide a macro perspective for understanding how abiotic stresses reshape the plant immune system.

In recent years, significant progress has been made in the study of the mechanisms by which tomatoes respond to low temperature stress. Research has indicated that CBF/DRE elements play a crucial role in cold stress [16]. Lin et al. found that *ICE1* can directly bind to the promoter of *CBF1*, and overexpressing *CaM6* at low temperatures can inhibit the transcriptional activity of *ICE1* to suppress the expression of *CBF1*, thereby reducing the cold tolerance of tomato plants [17]. The ICE1-CBF/DREB cascade is currently the most extensively studied regulatory pathway of cold stress response [18]. Studies have also shown that under cold stress, *SlMYB15* can directly bind to the *CBF* promoter and regulate its transcription, thereby enhancing its cold tolerance. Furthermore, *HY5* can regulate the transcription of *CBFs* directly or indirectly through *MYB15* in response to low temperature stress [19]. Under low temperature induction, *PIF4* enhances the low temperature resistance of tomato by upregulating the expression of *CBF1* [20]. Studies have shown that sub-low temperature can induce plant resistance [21,22,23]. Sub-low temperature can widely trigger acquired disease resistance in a variety of field and horticultural crops beyond tomato, mainly by activating plant hormone signal pathways and defense-related gene expression. Such temperature-induced resistance is conserved across different plant families and shows stable effects against fungi, bacteria and partial viruses [24,25]. Core cold-responsive transcription factors act as key molecular links connecting low temperature signals and plant immune responses [26]. At present, there is still a research gap in the application of sub-low temperature treatment in tomato resistance to TSWV. However, prolonged exposure of plants to moderate abiotic stress can also lead to conditions similar to those caused by abiotic stress. Therefore, to achieve the goal of inducing plant resistance through sub-low temperature treatment, it is crucial to control the duration of sub-low temperature exposure.

In this study, we employed a method involving sub-low temperature treatment followed by TSWV inoculation to investigate the impact of different sub-low temperature conditions on tomato resistance to TSWV. We validated the expression levels of *NSs* genes, *CBF* family genes, and hormone pathway-related genes in tomatoes through RT-qPCR. Furthermore, through transcriptome sequencing, we analyzed the key pathways involved in tomato disease resistance following sub-low temperature treatment. Overall, our research results expand the current understanding of additional pathways involved in tomato resistance to viral infection and provide a theoretical foundation and novel insights for enhancing tomato disease and stress resistance in the future.

## 2. Results

### 2.1. Sub-Low Temperature Pretreatment Correlates with Decreased TSWV Disease Severity in Tomato

**Figure 1:**
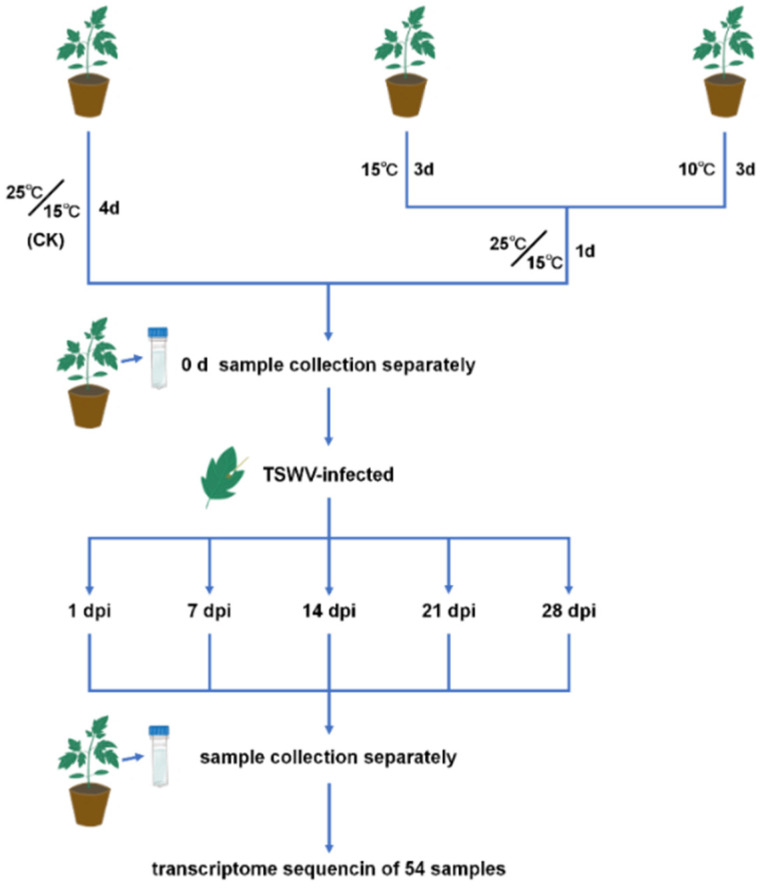
Schematic diagram of the experiment.

Tomato seedlings were pretreated with sub-low temperature regimes of 10 °C and 15 °C, with plants maintained at ambient temperature serving as the control group. Upon completion of cold priming, all experimental plants were mechanically inoculated uniformly with *Tomato spotted wilt virus* (TSWV). Subsequent disease development was monitored continuously over a 28-day period post-inoculation, and the corresponding results are presented in Figure 1.

**Figure 2:**
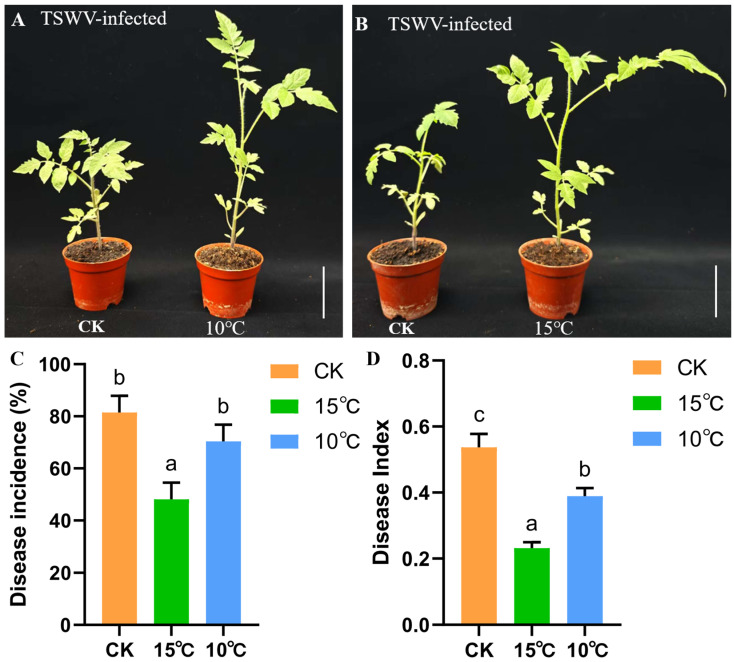
Phenotypes and disease incidence of tomato plants inoculated with TSWV under different temperature treatments. (**A**) Symptoms of tomato plants inoculated with TSWV at 28 days post-inoculation (dpi) in the room temperature control and 10 °C sub-low temperature treatment groups (scale bar = 10 cm). (**B**) Symptoms of tomato plants inoculated with TSWV at 28 dpi in the room temperature control and 15 °C sub-low temperature treatment groups (scale bar = 10 cm). (**C**) Comparison of disease incidence at 28 dpi among the room temperature control, 15 °C, and 10 °C sub-low temperature treatment groups. (**D**) Comparison of disease index at 28 dpi among the room temperature control, 15 °C, and 10 °C sub-low temperature treatment groups. The data of the three biological samples are expressed as the mean ± SD. Significance levels were marked with lowercase letters, where different letters above the bars indicate significant differences at *p* < 0.05.

The results revealed that tomato plants subjected to 10 °C and 15 °C sub-low temperature pretreatments exhibited milder TSWV disease phenotypes and lower disease severity relative to the 25 °C ambient temperature control (Figure 2A,B), a pattern correlated with cold priming. The control plants exhibited typical and severe symptoms following TSWV inoculation, including obvious chlorotic yellow spots and irregular necrotic lesions on newly emerged leaves. These plants also showed stunted growth and a weakened appearance. In contrast, the disease progression in tomato plants pretreated at 10 °C and 15 °C was markedly delayed. Only slight chlorotic dots and pale-yellow spots were observed on a few young leaves. These plants maintained a normal architecture with almost no signs of stunting and grew vigorously, indicating that all types of disease symptoms were significantly suppressed (Figure 2A,B). Disease incidence was statistically analyzed at 28 days post-inoculation (dpi). The results showed that the TSWV incidence in the 15 °C sub-low temperature treatment group was significantly lower than that in both the room temperature control and the 10 °C sub-low temperature treatment groups (Figure 2C). After treatments with sub-low temperatures of 15 °C and 10 °C, the disease indices of tomato plants were 23.15% and 38.89%, respectively. In contrast, the disease index of tomato plants under the normal temperature control reached 53.70% (Figure 2D). The disease indices of tomato plants subjected to sub-low temperature treatments were significantly lower than that of the control group. The recorded phenotypes show that 15 °C sub-low temperature conditioning correlates with lowered tomato spotted wilt disease incidence.

### 2.2. Sub-Low Temperature Priming Correlates with Reduced TSWV Accumulation in Tomato

**Figure 3:**
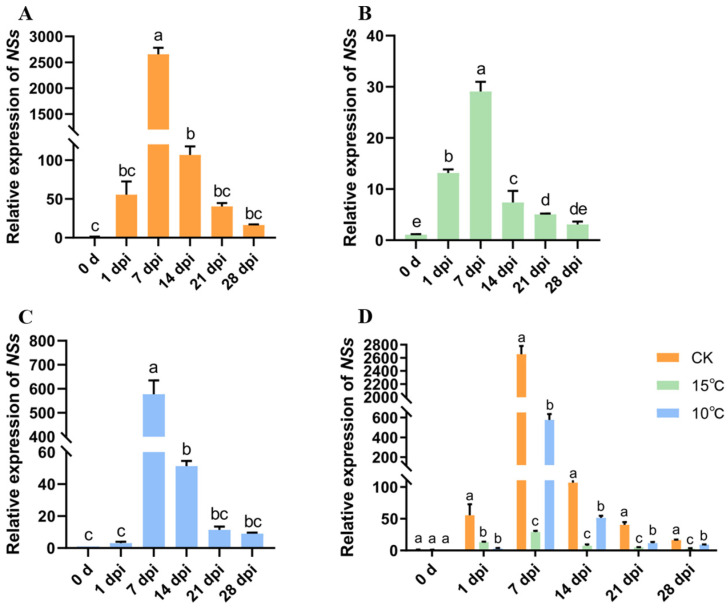
TSWV accumulation in tomato plants after different temperature treatments. (**A**) Dynamics of virus accumulation in tomato plants inoculated with TSWV without sub-low temperature treatment. (**B**) Dynamics of virus accumulation in tomato plants inoculated with TSWV after 15 °C sub-low temperature treatment. (**C**) Dynamics of virus accumulation in tomato plants inoculated with TSWV after 10 °C sub-low temperature treatment. (**D**) Comparison of virus accumulation among the untreated control and the two sub-low temperature treatment groups. The data of the three biological samples are expressed as the mean ± SD. Significance levels were marked with lowercase letters, where different letters above the bars indicate significant differences at *p* < 0.05.

The relative expression of the viral *NSs* gene, a proxy for TSWV accumulation, exhibited significant differences across tomato plants subjected to distinct temperature regimens (Figure 3). In the room temperature control group (CK), the expression of the *NSs* gene increased initially and then decreased over time post-inoculation (dpi), peaking at 7 dpi (approximately 2600-fold, significantly higher than at other time points, *p* < 0.05). Subsequently, it declined continuously from 14 to 28 dpi but remained significantly higher than the initial level (Figure 3A). In the 15 °C sub-low temperature group, the *NSs* gene expression also peaked at 7 dpi; however, the peak level (approximately 29-fold) was significantly lower than that of the CK group, with overall expression levels remaining in a low range and a decline in the later stages (Figure 3B). In the 10 °C sub-low temperature group, the *NSs* gene expression reached its peak at 7 dpi (approximately 580-fold), which was significantly higher than the levels at 14–28 dpi but remained far lower than that of the CK group (Figure 3C). A comprehensive comparison of the viral accumulation dynamics across the three groups (Figure 3D) revealed that *NSs* gene expression in the CK group was significantly higher throughout the observation period compared to both the 15 °C and 10 °C sub-low temperature groups, with particularly notable differences in peak values (approximately 2600-fold in CK vs. 29-fold and 580-fold in the 15 °C and 10 °C groups, respectively). Across all sampling time points, 10 °C and 15 °C sub-low temperature priming correlated with lower viral accumulation, with the 15 °C treatment displaying the most marked reduction in TSWV accumulation.

### 2.3. Expression Analysis of CBF Genes in Tomato Plants Infected with TSWV

**Figure 4:**
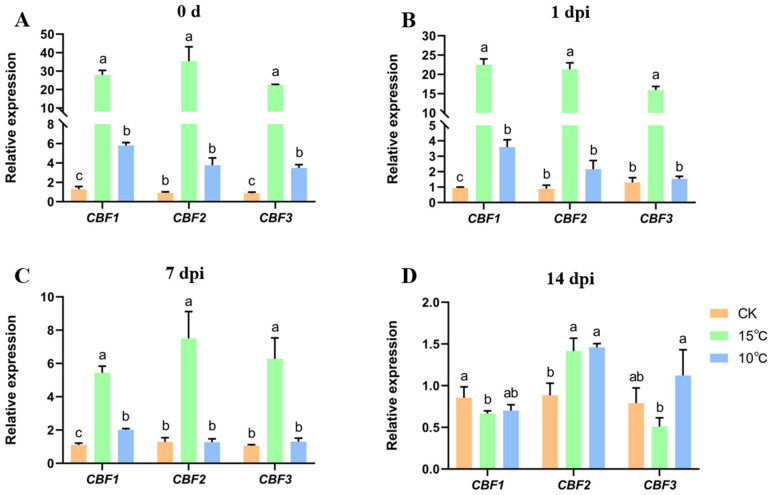
Expression patterns of cold-responsive CBF family genes (*CBF1*, *CBF2*, and *CBF3*). The data of the three biological samples are expressed as the mean ± SD. Significance levels were marked with lowercase letters, where different letters above the bars indicate significant differences at *p* < 0.05. (**A**) Expression patterns of cold-responsive CBF family genes (*CBF1*, *CBF2*, and *CBF3*) at 0 d; (**B**) Expression patterns of cold-responsive CBF family genes (*CBF1*, *CBF2*, and *CBF3*) at 1 dpi; (**C**) Expression patterns of cold-responsive CBF family genes (*CBF1*, *CBF2*, and *CBF3*) at 7 dpi; (**D**) Expression patterns of cold-responsive CBF family genes (*CBF1*, *CBF2*, and *CBF3*) at 14 dpi.

The expression patterns of cold-responsive CBF family genes (*CBF1*, *CBF2*, and *CBF3*) in tomato plants under different temperature treatments were analyzed at 0, 1, 7, and 14 days post-inoculation (dpi) (Figure 4A–D). At 0 d, prior to virus inoculation, both the 10 °C and 15 °C sub-low temperature treatments significantly upregulated the expression of all three *CBF* genes compared with the room-temperature control (CK). Notably, the 15 °C treatment induced a much stronger response, with the expression levels of *CBF1*, *CBF2*, and *CBF3* reaching 27.5-, 35.5-, and 22.5-fold higher than in CK, respectively, whereas the 10 °C treatment resulted in only moderate induction (approximately 5.7-, 3.5-, and 3.1-fold). This trend was maintained at 1 and 7 dpi, where the 15 °C group consistently showed significantly higher *CBF* expression than both the CK and 10 °C groups. By 14 dpi, however, the differences diminished; while the CK group exhibited stable expression, the induced *CBF* expression in both low-temperature treatments gradually declined to levels closer to the control, indicating a transient but robust activation of the cold response pathway. Notably, the magnitude and duration of *CBF* gene induction exhibited a tight correlation with the degree of TSWV accumulation suppression. The 15 °C treatment triggered the strongest and most persistent transcriptional activation of *CBFs*, which correlated with the lowest TSWV accumulation (Figure 3). This coordinated transcriptional pattern reveals an association between amplified *CBF* cold signaling and improved tomato performance against TSWV under sub-low temperature priming; however, this correlative expression evidence is insufficient to confirm a direct causal role of *CBF* genes in mediating antiviral resistance.

### 2.4. Changes in Differentially Expressed Genes Under Different Temperature Treatments

**Figure 5:**
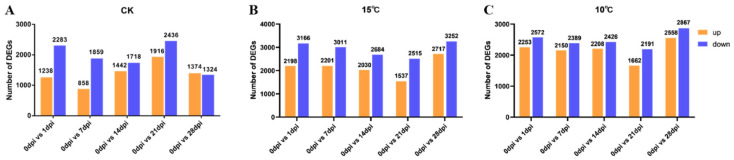
Number of differentially expressed genes after inoculation with TSWV under different temperature treatments. (**A**) Number of differentially expressed genes after inoculation with TSWV under 25 °C temperature treatment; (**B**) Number of differentially expressed genes after inoculation with TSWV under 15 °C temperature treatment; (**C**) Number of differentially expressed genes after inoculation with TSWV under 10 °C temperature treatment.

To characterize the global transcriptional reprogramming in tomato plants in response to TSWV infection under different temperature regimes, the number of differentially expressed genes (DEGs) at each time point post-inoculation (dpi) was quantified relative to the 0 dpi baseline (Figure 5). All differentially expressed genes identified herein represent combined transcriptional responses to sub-low temperature priming and subsequent TSWV inoculation, rather than virus-specific responses isolated from sub-low temperature stress effects.

In the room-temperature control (CK) group (Figure 5A), the transcriptional response to infection was relatively moderate. The number of upregulated genes reached a minimum of 858 at 7 dpi. This phenomenon corresponds to the middle stage of TSWV infection: viral proliferation and systemic spread gradually intensified at this time, which suppressed the normal defense activation of tomato plants and resulted in the lowest level of gene upregulation. Meanwhile, the number of downregulated genes peaked at 2436 at 21 dpi. Conversely, both sub-low temperature treatments elicited substantially stronger and more sustained transcriptional responses (Figure 5B,C). In the 15 °C treatment group (Figure 5B), the magnitude of DEGs was consistently higher than in the CK group across all time points, with the maximum number of upregulated (2717) and downregulated (3252) genes observed at 28 dpi. Similarly, the 10 °C treatment (Figure 5C) also induced a robust transcriptional response, particularly in the later stages of infection, peaking at 28 dpi with 2558 upregulated and 2867 downregulated genes. The peak of DEGs at 28 dpi in the two sub-low temperature groups occurred at the late infection stage when typical viral symptoms fully developed. The pre-induced cold response pathways continuously coordinated with antiviral defense pathways, triggering persistent and intense transcriptional reprogramming to resist sustained viral invasion, thus leading to the maximum number of differentially expressed genes at this time point.

Across all sampling points, the number of both upregulated and downregulated DEGs was markedly higher in the 15 °C and 10 °C groups compared to the CK group. Notably, the most pronounced transcriptomic reprogramming occurred under the 15 °C treatment, which correlated with reduced TSWV accumulation and alleviated disease symptoms. These transcriptional profiles reveal that sub-low temperature preconditioning, particularly the 15 °C regimen, correlates with broader and more sustained transcriptomic remodeling in tomato upon TSWV inoculation.

### 2.5. Enriched Pathways of Differentially Expressed Genes

**Figure 6:**
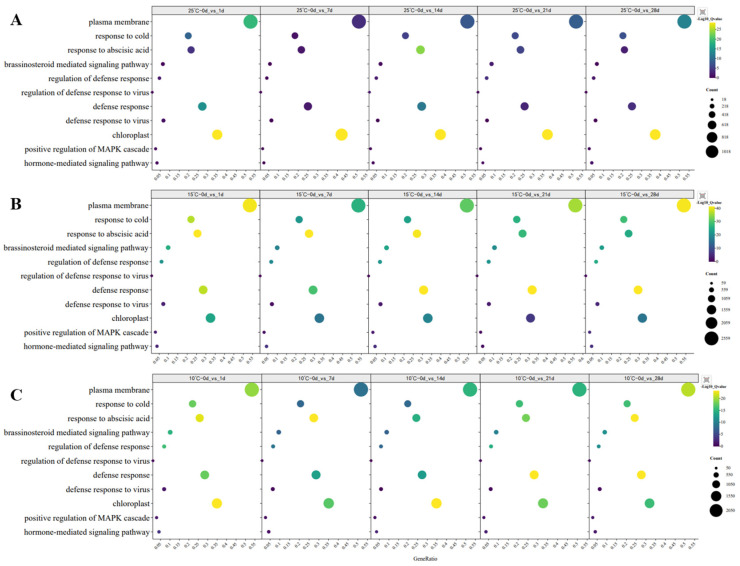
Pathways enriched by differentially expressed genes. (**A**) Pathways enriched by differentially expressed genes under 25 °C temperature treatment; (**B**) Pathways enriched by differentially expressed genes under 15 °C temperature treatment; (**C**) Pathways enriched by differentially expressed genes under 10 °C temperature treatment.

To further characterize the functional relevance of temperature-modulated transcriptomic responses, Gene Ontology (GO) enrichment analysis was performed on the DEGs from the CK (25 °C), 15 °C, and 10 °C groups across different infection stages (Figure 6A–C). A total of 11 core biological processes were consistently evaluated, including plasma membrane, response to cold, response to abscisic acid, brassinosteroid-mediated signaling pathway, regulation of defense response, regulation of defense response to virus, defense response, defense response to virus, chloroplast, positive regulation of MAPK cascade, and hormone-mediated signaling pathway.

In the CK group (Figure 6A), the enrichment of defense-related and cold-responsive pathways was relatively weak, with most terms showing low GeneRatio and enrichment scores across all time points. In contrast, both sub-low temperature treatments significantly enhanced the enrichment of these pathways. The 15 °C treatment (Figure 6B) exhibited the most pronounced and consistent enrichment of key functional categories, particularly response to cold, defense response to virus, and brassinosteroid-mediated signaling pathway, which displayed high GeneRatio, large gene counts, and significant enrichment (0) throughout the infection period. The 10 °C group (Figure 6C) also showed enrichment of these pathways, though generally with lower magnitude and consistency compared to the 15 °C group.

This difference is attributed to the distinct physiological and molecular thresholds between 15 °C moderate sub-low temperature and 10 °C relatively extreme sub-low temperature. As a mild cold condition, 15 °C acts as an effective priming stimulus: it sufficiently activates cold-responsive cascades and antiviral signaling networks without triggering severe cold stress. The coordinated activation of cold acclimation and defense pathways can be stably maintained, thereby sustaining strong functional pathway enrichment and robust antiviral performance. In contrast, 10 °C represents an extreme sub-low temperature beyond the optimal priming threshold for tomato. Although it can induce cold and defense-related pathways to a certain extent, this temperature imposes additional cold stress on plants. Excess cold pressure partially suppresses the normal operation of immune signaling, disrupts the synergism between cold response and antiviral pathways, and weakens the continuity and intensity of pathway enrichment. At the physiological level, 10 °C impairs cell membrane stability and cellular homeostasis to a certain degree; at the molecular level, it disturbs the balanced expression of functional genes. Collectively, these threshold differences make 15 °C more efficient in inducing plant antiviral resistance than 10 °C.

Notably, pathways for antiviral defense and cold acclimation displayed the strongest co-enrichment in the 15 °C group, correlating with the lower viral accumulation and milder disease symptoms documented above. These transcriptomic data identify a correlation between 15 °C preconditioning and the coordinated induction of cold and antiviral signaling programs, a molecular signature putatively linked to less severe TSWV infection in tomato.

**Figure 7:**
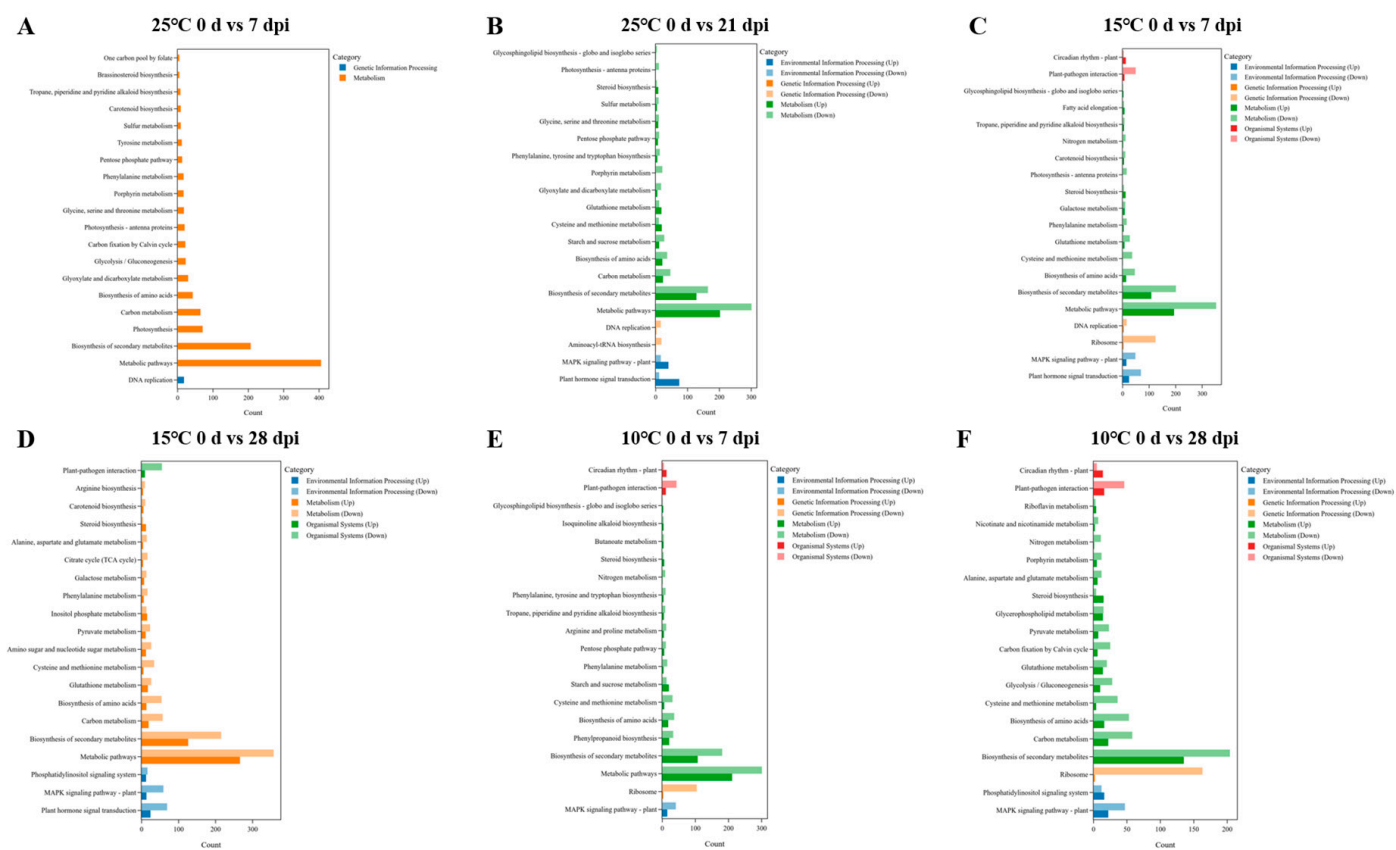
KEGG pathway enrichment analysis of DEGs. In the KEGG enrichment bar plot, the *x*-axis shows pathway names, and the *y*-axis shows the number of enriched genes, which reflects the distribution and enrichment degree of functional pathways. (**A**) KEGG pathway enrichment analysis of DEGs between the 25 °C 0 d and 7 dpi conditions; (**B**) KEGG pathway enrichment analysis of DEGs between the 25 °C 0 d and 21 dpi conditions; (**C**) KEGG pathway enrichment analysis of DEGs between the 15 °C 0 d and 7 dpi conditions; (**D**) KEGG pathway enrichment analysis of DEGs between the 15 °C 0 d and 28 dpi conditions; (**E**) KEGG pathway enrichment analysis of DEGs between the 10 °C 0 d and 7 dpi conditions; (**F**) KEGG pathway enrichment analysis of DEGs between the 10 °C 0 d and 28 dpi conditions.

To comprehensively dissect the functional pathways modulated by tomato transcriptome in response to TSWV infection under different temperature 25 °C, 15 °C, and 10 °C conditions, we performed KEGG pathway enrichment analysis on DEGs at key infection stages. As shown in Figure 7A–F, the enriched pathways were classified into four major functional categories: Metabolism, Genetic Information Processing, Environmental Information Processing, and Organismal Systems, with distinct enrichment patterns shaped by both temperature and infection progression. Under the control 25 °C temperature, the enrichment landscape was dominated by metabolic pathways at both 7 dpi early infection stages (Figure 7A) and 21 dpi late infection stages (Figure 7B). At 7 dpi, core metabolic processes including “Metabolic pathways” and “Biosynthesis of secondary metabolites” harbored the highest number of DEGs, with additional enrichment in carbon metabolism, photosynthesis, and amino acid biosynthesis pathways, reflecting the plant’s basal metabolic reprogramming triggered by viral infection. By 21 dpi, while metabolic pathways remained predominant, signaling pathways such as “Plant hormone signal transduction” and “MAPK signaling pathway—plant” began to show moderate enrichment, indicating a gradual activation of stress signaling cascades under normal temperature conditions.

In contrast, 15 °C and 10 °C sub-low temperature conditions significantly reshaped the KEGG enrichment profiles, with a pronounced enhancement of both cold-responsive metabolic pathways and antiviral defense-related signaling pathways (Figure 7C–F). At 15 °C, “Plant–pathogen interaction”—a key pathway mediating plant innate immunity against viral invasion—was significantly enriched at both 7 dpi and 28 dpi, with the number of associated DEGs increasing notably at the 28 dpi later stage (Figure 7D). Concurrently, cold-adaptive metabolic pathways, including “Starch and sucrose metabolism”, “Glycolysis/Gluconeogenesis”, and “Carbon metabolism”, showed robust enrichment, suggesting that the combined stress of low temperature and viral infection triggered coordinated reprogramming of primary metabolism to maintain cellular homeostasis. At the 10 °C temperature, this trend was further amplified: both “Plant–pathogen interaction” and “Circadian rhythm—plant” pathways were prominently enriched at both 7 dpi early infection stages (Figure 7E) and 28 dpi late infection stages (Figure 7F), indicating persistent activation of antiviral defense and temperature-sensitive regulatory networks. Across all sub-low temperature groups, signaling pathways such as “MAPK signaling pathway—plant” and “Plant hormone signal transduction” were consistently enriched, with stronger and more sustained activation compared to the 25 °C control. Collectively, these transcriptomic profiles reveal an association between sub-low temperature conditioning and reshaped tomato transcriptional responses to TSWV infection. The cold regimen correlates with amplified antiviral defense pathway activation alongside altered metabolic homeostasis, a molecular signature putatively linked to coordinated shifts in central metabolism and immune signaling.

**Figure 8:**
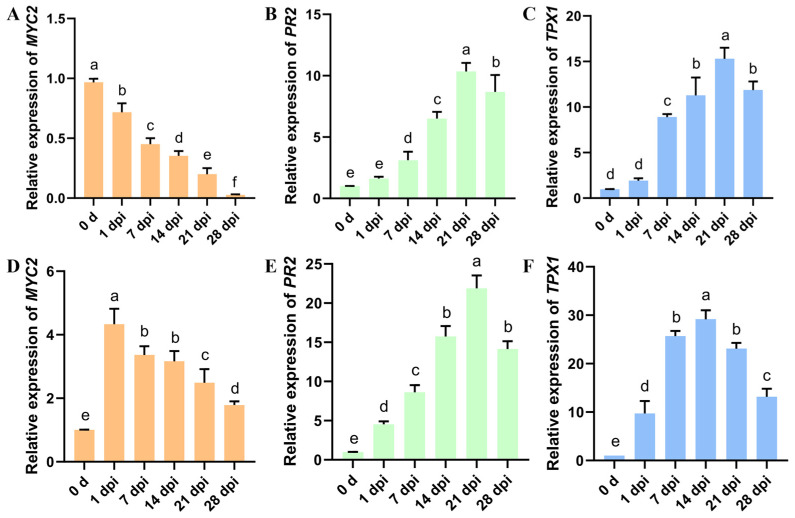
Expression patterns of *MYC2*, *PR2* and *TPX1* in TSWV-infected tomato plants grown at different temperatures. (**A**) *MYC2* expression dynamics in TSWV-infected tomato plants grown at normal temperature. (**B**) *PR2* expression dynamics in TSWV-infected tomato plants grown at normal temperature. (**C**) *TPX1* expression dynamics in TSWV-infected tomato plants grown at normal temperature. (**D**) Expression dynamics of *MYC2* in tomato plants after 15 °C sub-low temperature pretreatment and subsequent TSWV inoculation. (**E**) Expression dynamics of *PR2* in tomato plants after 15 °C sub-low temperature pretreatment and subsequent TSWV inoculation. (**F**) Expression dynamics of *TPX1* in tomato plants after 15 °C sub-low temperature pretreatment and subsequent TSWV inoculation. The data of the three biological samples are expressed as the mean ± SD. Significance levels were marked with lowercase letters, where different letters above the bars indicate significant differences at *p* < 0.05.

RT-qPCR was performed to characterize the time-series expression of *MYC2*, *PR2* and *TPX1* in TSWV-infected tomatoes with or without 15 °C sub-low temperature pretreatment (Figure 8). At normal temperature, TSWV infection persistently suppressed *MYC2* expression over 0–28 dpi, while inducing continuous upregulation of *PR2* and *TPX1*, which reached maximum expression at 21 dpi. After 15 °C cold pre-treatment, the basal abundance of *MYC2* was greatly improved; although TSWV infection triggered a gradual reduction in *MYC2* after an early sharp rise at 1 dpi, its expression was always higher than that in cold-untreated infected plants. In addition, cold pre-treatment synergistically amplified the induction of *PR2* and *TPX1* upon TSWV inoculation, resulting in much higher peak expression at 21 dpi than the normal-temperature group. The expression trends of the three genes determined by RT-qPCR were fully consistent with the transcriptome results, confirming the authenticity of RNA-seq-derived transcriptional responses.

## 3. Discussion

*Tomato spotted wilt virus* (TSWV) currently stands as one of the most devastating pathogens in global tomato production, posing severe threats to food security and the agricultural economy. The management of this disease is notably complex and challenging, primarily due to its extensive host range and its transmission by thrips vectors [27,28]. Contemporary management strategies rely on an integrated approach that encompasses the deployment of resistant varieties carrying the *Sw-5* or *Sw-7* genes, rigorous vector control using selective insecticides and biocontrol agents, as well as the implementation of cultural and sanitary practices to eliminate viral reservoirs. Despite these advancements, the emergence of resistance-breaking strains and the continuous adaptation of vector populations persist as critical challenges to the sustainability of TSWV management in tomato crops [29]. In this context, our phenotypic and transcriptomic data reveal a correlation between sub-low temperature conditioning (notably 15 °C) and reduced TSWV accumulation in tomato. Differential effects on tomato antiviral resistance were observed between 10 °C and 15 °C. Severe cold stress at 10 °C disturbs primary metabolism and damages cellular homeostasis, ultimately weakening plant defense ability. As a sub-low temperature matching tomato growth environment, 15 °C effectively initiates cold-responsive and defense-related signaling pathways, and further strengthens antiviral resistance without inducing cold injury. This explains why 15 °C serves as a more favorable condition for inducing plant disease resistance. This 15 °C preconditioning regimen may hold hypothetical promise for TSWV-susceptible tomato crops in solar plastic greenhouses. Ventilation and shade nets could theoretically maintain this mild low-temperature regime with little extra cost, yet comprehensive agronomic trials and formal economic evaluations to verify its on-site utility are absent. Although laboratory phenotypic and transcriptomic data link cold priming to reduced TSWV accumulation—an outcome that might hypothetically cut pesticide usage, labor costs and virus-induced yield losses—this strategy remains only an early laboratory-based concept. Multi-season field trials integrated with yield measurement and full cost–benefit economic analysis are mandatory to verify its practical efficacy and potential economic gains prior to any commercial greenhouse application.

Exposure to moderate abiotic stress stimuli can activate multiple intrinsic defensive signaling cascades within plant cells, thereby substantially elevating host resistance against a broad spectrum of destructive biotic stresses including viral pathogens [30,31,32]. In this study, we observed that the 15 °C pre-treatment induced a more robust and sustained upregulation of *CBF1*, *CBF2*, and *CBF3* compared to the 10 °C treatment. This molecular response correlated with significantly reduced accumulation of TSWV and milder disease symptoms in the 15 °C group. These observations reveal a correlative link between intensified activation of the *CBF*-dependent cold cascade and improved tomato performance against TSWV under sub-low temperature treatments, yet our current transcriptomic data cannot establish a direct causal relationship between *CBF* induction and enhanced viral tolerance. We propose several potential mechanisms that may explain this observed association. First, activated *CBF* signaling drives the transcription of downstream cold-responsive *COR* genes, whose encoded products function in cryoprotection, antioxidant homeostasis and cell wall remodeling. Such cellular protective processes could alleviate stress-induced damage, indirectly limiting TSWV replication and systemic movement [33]. Second, cold-triggered *CBF* signaling may engage in molecular crosstalk with antiviral phytohormone cascades, including salicylic acid (SA), jasmonic acid (JA) and ethylene (ET) pathways [24,25,34]. Concurrent upregulation of *CBF* transcripts may transcriptionally prime these immune branches to mount a more robust defensive response upon TSWV challenge, though genetic functional validation is still required to verify this regulatory connection.

Transcriptomic analysis revealed that both sub-low temperature pre-treatments, particularly at 15 °C, induced a more extensive transcriptional reprogramming in TSWV-infected tomato plants compared to the room temperature control. The elevated number of DEGs observed in the 15 °C pre-treated samples coincided with the suppression of viral accumulation. This broad alteration in the number of up- and down-regulated genes likely reflects a coordinated response to both cold stress and viral infection. GO enrichment analysis and KEGG pathway enrichment analysis further elucidated the functional basis for the enhanced antiviral tolerance observed under sub-low temperature conditions. Compared to the room temperature control, both the 15 °C and 10 °C treatments elicited stronger and more consistent enrichment of pathways associated with cold acclimation, hormone signaling, and antiviral defense. Notably, the 15 °C pretreatment displayed the broadest and most persistent enrichment of these core functional pathways, a molecular signature that correlates with diminished viral transcript abundance and reduced disease phenotypes observed in this group. These findings suggest that sub-low temperature pre-treatment activates a coordinated transcriptional network that links cold acclimation, hormone signaling, and antiviral immunity. Physiologically and cytologically, cold acclimation sustains intracellular homeostasis via osmotic adjustment, antioxidant reinforcement and cell membrane stabilization, which inhibits intracellular viral accumulation. Moreover, cold-triggered modifications to cell walls and plasmodesmata hinder the intercellular trafficking of viruses. Co-enrichment of cold and defense pathways is putatively linked to heightened plant responsiveness to TSWV, correlating with lower viral loads and limited systemic viral spread. In summary, sub-low temperature pretreatment correlates with improved tomato performance upon TSWV inoculation, an observation associated with cold-inducible transcriptional networks that overlap with antiviral immune cascades. Notably, the lack of sub-low temperature matched mock-inoculated controls limits our ability to decouple the independent transcriptional signatures elicited by sub-low temperature and TSWV infection. Accordingly, all transcriptomic interpretations presented herein are contextualized as integrated responses to concurrent cold priming and viral stress.

In summary, this study employed differential gene expression enrichment analysis to investigate tomato plants inoculated with TSWV following two sub-low temperature pre-treatments. Using room temperature-grown, TSWV-inoculated plants as a control, we identified key metabolic pathways underlying the tomato defense response after sub-low temperature exposure, providing valuable insights into novel mechanisms of TSWV resistance. However, several questions warrant further investigation. Firstly, this study adopted only one commercial tomato cultivar, which may reduce the general applicability of the results. For subsequent verification, we will select tomato cultivars with different TSWV resistance levels, apply 15 °C sub-low temperature pretreatment and TSWV inoculation, and compare their phenotypic and transcriptomic differences to confirm the conserved function of core antiviral pathways. Secondly, we experimentally confirmed the transcriptional patterns of hormone pathway-related genes within this study. Nevertheless, the master regulatory genes that orchestrate cold-triggered antiviral responses are yet to be functionally dissected. We will carry out gene overexpression and silencing experiments to characterize their biological roles and further clarify the molecular connection between cold response and viral resistance. Thirdly, the inability to separate cold-specific and virus-specific transcriptional responses is a key constraint of this work, and we propose parallel mock inoculation under each temperature as a priority direction for subsequent mechanistic verification. Fourthly, numerous studies indicate that temperature variations can enhance plant resistance to various diseases by recruiting beneficial microbes. For instance, heat stress-induced nucleotide enrichment has been shown to recruit beneficial rhizosphere microbiota, protecting plants against heat stress and root rot [35]. We will further investigate whether sub-low temperature modulates rhizosphere microbial communities to strengthen tomato resistance against TSWV. Rhizosphere samples from each treatment will be used for microbial high-throughput sequencing to analyze community structure and screen functional microorganisms associated with antiviral activity. In the future, integrated research on cultivar expansion, key gene function and rhizosphere microbiome will systematically elaborate the mechanism of sub-low temperature mediated antiviral resistance. It will also promote the popularization and application of sub-low temperature induction technology in tomato production.

## 4. Materials and Methods

### 4.1. Sub-Low Temperature Treatment of Tomato Plants

Uniform and plump seeds of the tomato cultivar ‘Jinhong Dijia Wang F1’ (Xi’an Jinsheng Seed Industry Co., Ltd., Xi’an, China) were sown in 98-cell plug trays filled with commercial professional peat substrate (Greenterra, Latvia) and raised using standard seedling cultivation practices. When seedlings grew to the two-true-leaf stage with the third leaf newly unfolded, they were placed in an artificial climate chamber for low-temperature treatment.

Three temperature groups were established: constant 15 °C (day/night), constant 10 °C (day/night), and the control group at 25 °C (day)/15 °C (night). All groups shared a 12 h/12 h photoperiod, 3000 lx light intensity and 60 ± 5% relative humidity. The sub-low temperature treatment was conducted for 3 days. This treatment duration was optimized according to our preliminary trials: it can effectively trigger cold acclimation and antiviral priming in tomato without inducing cold injury. Subsequently, seedlings were recovered for 1 day in the climate chamber under the control temperature, 3000 lx light intensity and 60 ± 5% relative humidity. The 1-day recovery enabled seedlings to recover from transient cold stress and reach a stable physiological state prior to inoculation. Afterwards, seedlings with consistent growth performance from each group were selected for TSWV inoculation.

### 4.2. TSWV Inoculation and Disease Incidence Statistics in Tomato Plants

Tomato seedlings subjected to different temperature treatments were inoculated with TSWV using a standardized mechanical inoculation method. TSWV-infected tomato leaf tissues stored at −80 °C were homogenized in 0.01 M phosphate-buffered saline (PBS, pH 7.4) at a ratio of 1:10 (*w*/*v*), supplemented with 1% (*w*/*v*) 600-mesh carborundum as an abrasive. The resulting viral sap was mechanically inoculated onto the fully expanded first true leaf of each seedling, with a consistent inoculum volume of 10 μL per plant. To ensure uniform inoculation pressure, the sap was gently spread over the entire leaf surface using a sterile cotton swab. After 5 min, inoculated leaves were rinsed with sterile deionized water to remove residual sap and carborundum. Seedlings were inoculated with TSWV following their respective temperature treatments (control, 15 °C, and 10 °C) and a 1-day recovery period at room temperature.

### 4.3. Disease Incidence and Disease Severity Index of Tomato Viral Symptoms

Disease symptoms were monitored daily, and disease incidence was calculated at 28 dpi as the percentage of plants exhibiting typical TSWV symptoms relative to the total number of inoculated plants. Phenotypic characterization and disease incidence analysis were performed with three independent biological replicates, each consisting of 27 tomato plants to ensure data robustness and reproducibility. Disease incidence (%) = (Number of diseased plants/Total surveyed plants) × 100. Disease resistance identification grading criteria and calculation method for disease severity index were counted using the method reported by Ma et al. [36].

### 4.4. Sample Collection

Following sub-low temperature treatment and subsequent recovery at room temperature, leaf samples were collected from all treatment and control groups. The sampling immediately after recovery was defined as 0 d. For all sampling events, non-inoculated leaves were used. Specifically, the top leaf on the first branch above the petiole of the inoculated leaf was collected at 0 d. For the subsequent time points (1, 7, 14, 21 and 28 dpi), samples were sequentially taken from newly developed branches upward along the main stem. Uniform leaves at the equivalent position of different branches were selected to guarantee sampling consistency.

The time series of 0 d, 1, 7, 14, 21 and 28 dpi was designed according to the infection characteristics of TSWV in tomato: 0d represented the state before inoculation with the virus; 1 dpi reflected the early stage of viral invasion; 7 and 14 dpi corresponded to the middle stage of viral accumulation and movement; 21 and 28 dpi covered the late infection stage when typical disease symptoms fully developed. This time gradient enabled continuous monitoring of dynamic changes in host response and viral accumulation throughout the whole infection process. Three independent biological replicates were performed in this experiment, with nine individual tomato plants examined for each replicate. A schematic diagram of the overall experimental procedure is presented in Figure 1.

### 4.5. RNA Extraction and RT-qPCR Analysis

Total RNA was isolated from different tomato leaf samples using TRIzol reagent (Invitrogen, Carlsbad, CA, USA) following the manufacturer’s protocols. The concentration of each RNA sample was quantified, and all samples were uniformly adjusted to 500 ng/μL. Subsequently, 2 μg of total RNA was reverse-transcribed into cDNA using the Takara PrimeScript™ RT reagent Kit with gDNA Eraser. RT-qPCR was carried out using SYBR Premix mix (Takara, Japan). The 20 μL reaction system contained 10 μL SYBR Premix mix, 0.4 μL forward primer (10 μM), 0.4 μL reverse primer (10 μM), 2 μL diluted cDNA template and 7.2 μL nuclease-free water. The amplification procedure was set as follows: initial denaturation at 95 °C for 30 s; followed by 40 cycles of 95 °C for 5 s and 60 °C for 30 s; a final melting curve stage at 95 °C for 15 s, 60 °C for 1 min and 95 °C for 15 s. Tomato *Actin* was selected as the internal reference gene for data normalization. The relative expression levels of NSs, CBF family genes and hormone pathway genes were calculated via the 2^−ΔΔ^*^Ct^* method [37]. Standard curves were generated with serially diluted cDNA to calculate amplification efficiency for each primer pair. Melt-curve analysis was performed after each qPCR run to confirm specific amplification of target fragments and eliminate interference from primer dimers and non-specific amplicons. Three biological replicates and four technical replicates were used for each treatment. Each biological sample was a mixture of leaves collected from nine individual plants. The sequences of all primers applied in this experiment are summarized in Table S1.

### 4.6. Transcriptome Sequencing and Data Processing

Total RNA was extracted from tomato leaves using TRIzol reagent (Invitrogen), and three independent biological replicates were prepared for each sample. The concentration and purity of total RNA were evaluated using an Implen NanoPhotometer^® ^(Implen GmbH, Munich, Germany). cDNA library construction and high-throughput transcriptome sequencing were performed by Beijing QinKe Biotechnology Co., Ltd. (Beijing, China) on the Illumina NovaSeq 6000 platform with 150 bp paired-end reads. Raw FASTQ reads were processed with fastp [38] for quality control and filtering. The detailed filtering standards were as follows: adapter sequences were removed; reads containing more than 50% low-quality bases (Q ≤ 20) and reads with over 10% ambiguous bases (N) were discarded. The resulting clean reads were mapped to the tomato reference genome SL3.0 (ITAG3.2) using HISAT2 [39]. Differentially expressed genes (DEGs) were identified with DESeq2, using the thresholds of adjusted *p*-value (p_adj) < 0.05 and |log_2_FC| > 1. Expression data were normalized via Z-score transformation in R (v3.2.0), and a heatmap was generated to visualize the expression patterns of DEGs across different groups. Gene Ontology (GO) enrichment analysis was conducted on the DEGs using the TBtools (TBtools-II) to predict their potential biological functions. KEGG pathway enrichment analysis was performed to identify functional pathways enriched among differentially expressed genes. All *p*-values obtained from GO and KEGG enrichment analyses were corrected via the Benjamini–Hochberg false discovery rate algorithm to address multiple testing bias. Terms with adjusted *p*-values less than 0.05 were defined as significantly enriched functional categories. All raw RNA-seq sequencing data generated in this study have been deposited in the NCBI Sequence Read Archive (SRA) under accession number PRJNA1482784. RNA-seq quality control; PCA components; transcriptome expression boxplots; clustering results; volcano plots and comprehensive gene expression heatmaps of differentially expressed genes are presented in the supplementary files, including Supplementary Figures S1–S3.

### 4.7. Statistical Analysis

Three biological replicates and four technical replicates were used for each treatment. The data of the three biological samples are expressed as the mean ± SD. The significant difference between the control and treatment groups in each experiment was determined by one-way analysis of variance (ANOVA) followed by Duncan’s multiple range test. Significance levels were marked with lowercase letters, where different letters above the bars indicate significant differences at *p* < 0.05. All analyses were performed using IBM SPSS Statistics 27 and GraphPad Prism 9.

## Data Availability

The original contributions presented in this study are included in the article/Supplementary Materials. Further inquiries can be directed to the corresponding author.

## Supplementary Materials

The following supporting information can be downloaded at: https://www.mdpi.com/article/10.3390/plants15132058/s1, Table S1. Primer information. Supplementary Figure S1. Transcriptome boxplot. Supplementary Figure S2. DEGs Volcano plots. Supplementary Figure S3. DEGs pheatmap plots.
