# Supplementary material for: Sub-Low Temperature Preconditioning Induced Cold Signaling and Antiviral Defenses Correlate with Reduced TSWV Accumulation in Tomato
Source: Plants (Basel). 2026 Jul 2;15(13):2058. doi: 10.3390/plants15132058 (PMC13363850; doi:10.3390/plants15132058)
Supplement: Supplementary file 1 [file plants-15-02058-s001.zip › plants-4361546-table S1.pdf]

Table S1.Primer information

Primer name	Sequence (from 5' to 3')
**ACTIN-qF**	GTCCTCTTCCAGCCATCCAT
**ACTIN-qR**	ACCACTGAGCACAATGTTACCG
**TSWV-NSs-qF**	GTCTCCTGCTCAGCTCCATTC
**TSWV-NSs-qR**	TTTCTTGGAGCTGGAATCGGT
**CBF1-qF**	GTCATCGTCGTTTTCTGAAG
**CBF1-qR**	AACGGCCTCTTAATGCTAAA
**CBF2-qF**	TTCGATCGGAAGAAGTTTCA
**CBF2-qR**	CAAGTAATCCTGGCATGGAA
**CBF3-qF**	CGCCGAAATCTTCCGACCT
**CBF3-qR**	CGGCATGCAGAATAACGCTT
**MYC2-qF**	GTGCTTCCAGTGCCAATGTG
**MYC2-qR**	GCCCGAAGAAGGCAAAACTG
**PR2-qF**	GGACACCCTTCCGCTACTCTT
**PR2-qR**	TGTTCCTGCCCCTCCTTTC
**TPX1-qF**	GAGATGCAGTTGTGGCTACG
**TPX1-qR**	GCGAAGGATTGTTGCAGTCT

Purpose
RT-qPCR for the reference gene ACTIN in tomato
RT-qPCR for the reference gene ACTIN in tomato
RT-qPCR for validation of the TSWV-NSs transcription level
RT-qPCR for validation of the TSWV-NSs transcription level
RT-qPCR for validation of the CBF1 transcription level
RT-qPCR for validation of the CBF1 transcription level
RT-qPCR for validation of the CBF2 transcription level
RT-qPCR for validation of the CBF2 transcription level
RT-qPCR for validation of the CBF3 transcription level
RT-qPCR for validation of the CBF3 transcription level
RT-qPCR for validation of the MYC2 transcription level
RT-qPCR for validation of the MYC2 transcription level
RT-qPCR for validation of the PR2 transcription level
RT-qPCR for validation of the PR2 transcription level
RT-qPCR for validation of the TPX1 transcription level
RT-qPCR for validation of the TPX1 transcription level
