# Supplementary material for: Sub-Low Temperature Preconditioning Induced Cold Signaling and Antiviral Defenses Correlate with Reduced TSWV Accumulation in Tomato
Source: Plants (Basel). 2026 Jul 2;15(13):2058. doi: 10.3390/plants15132058 (PMC13363850; doi:10.3390/plants15132058)
Supplement: Supplementary file 1 [file plants-15-02058-s001.zip › Supplementary Figures.pptx]

## Slide 1
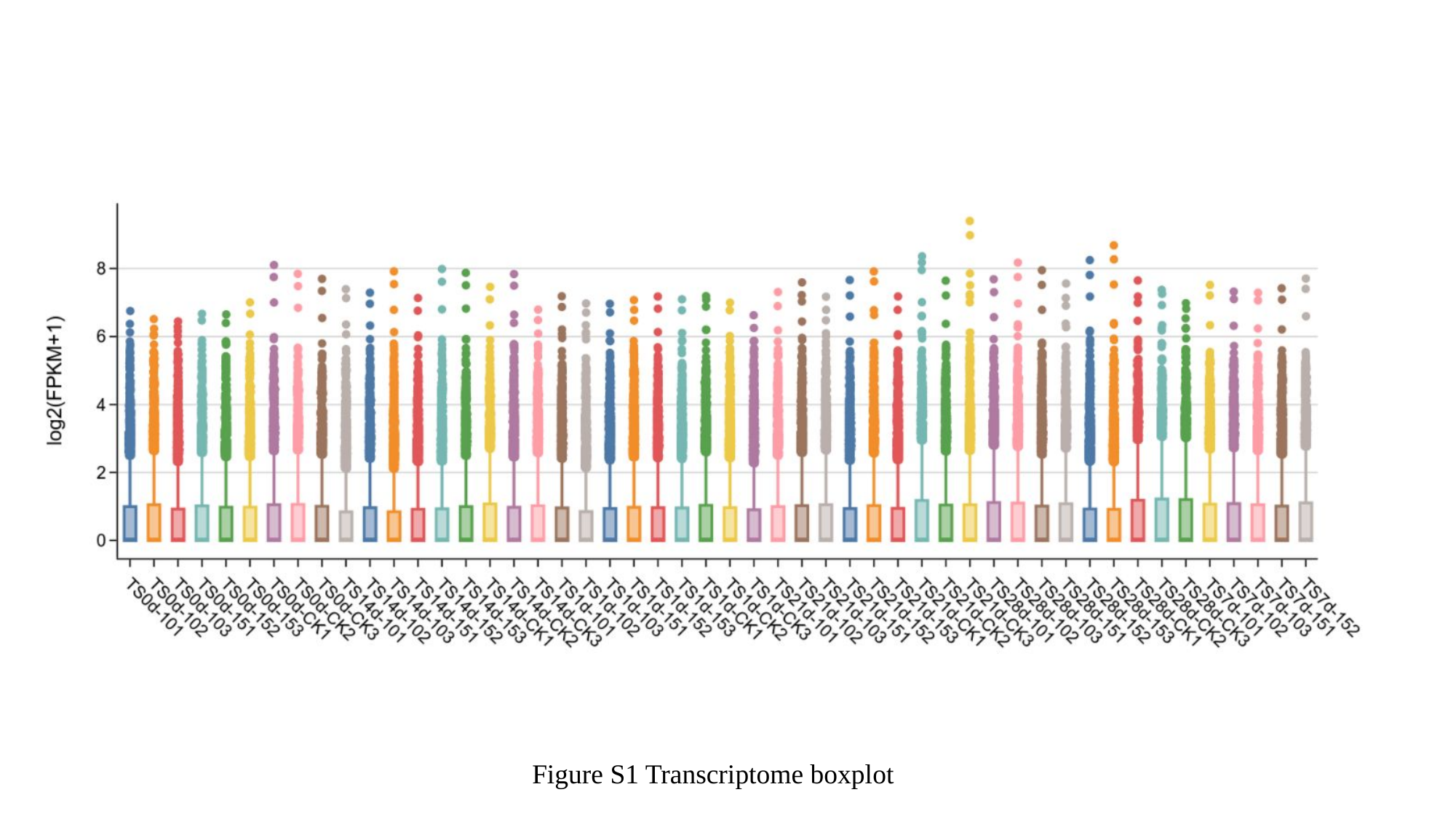

Figure S1 Transcriptome boxplot

## Slide 2
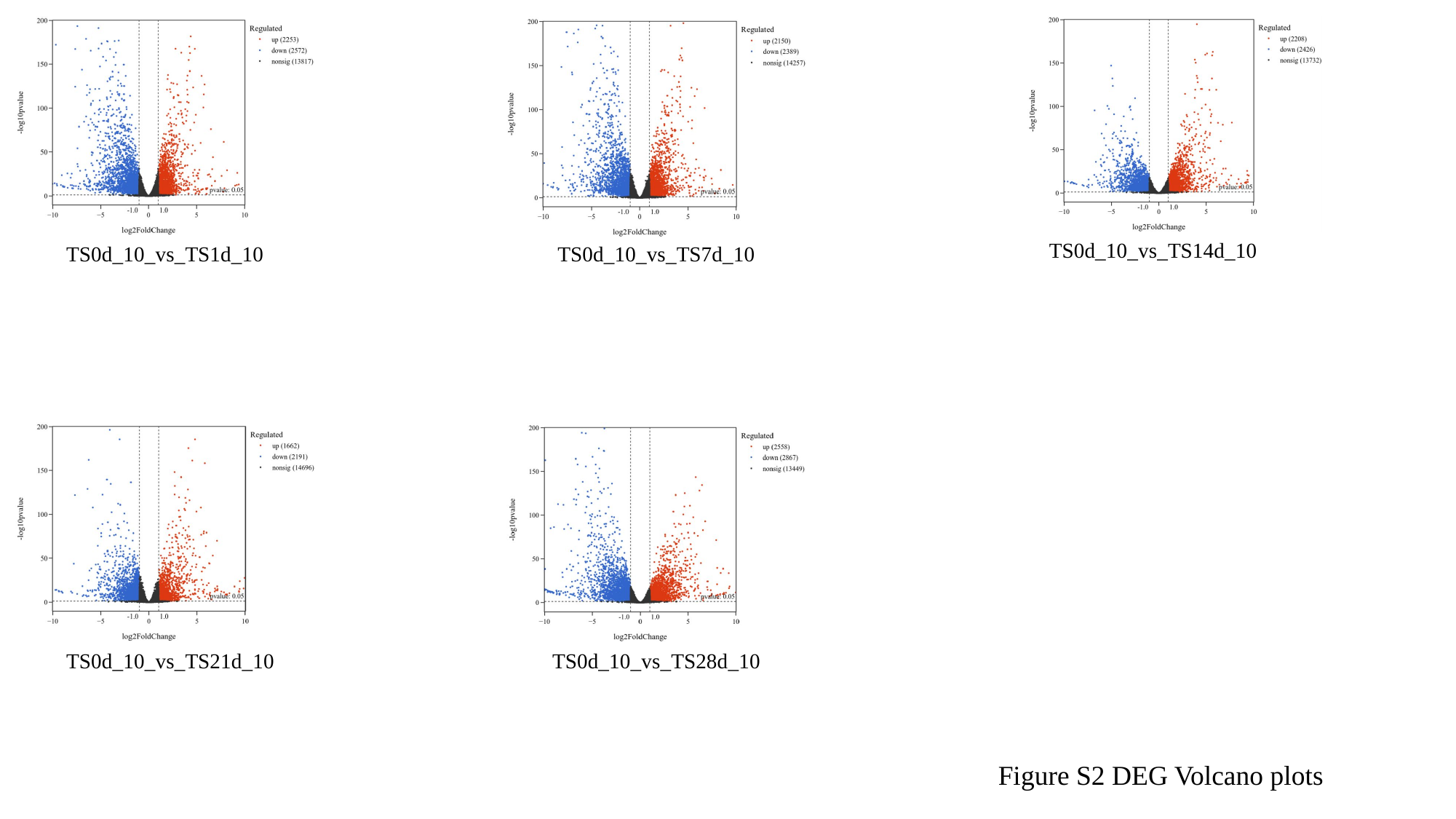

TS0d_10_vs_TS14d_10
TS0d_10_vs_TS7d_10
TS0d_10_vs_TS1d_10
TS0d_10_vs_TS28d_10
TS0d_10_vs_TS21d_10
Figure S2 DEG Volcano plots

## Slide 3
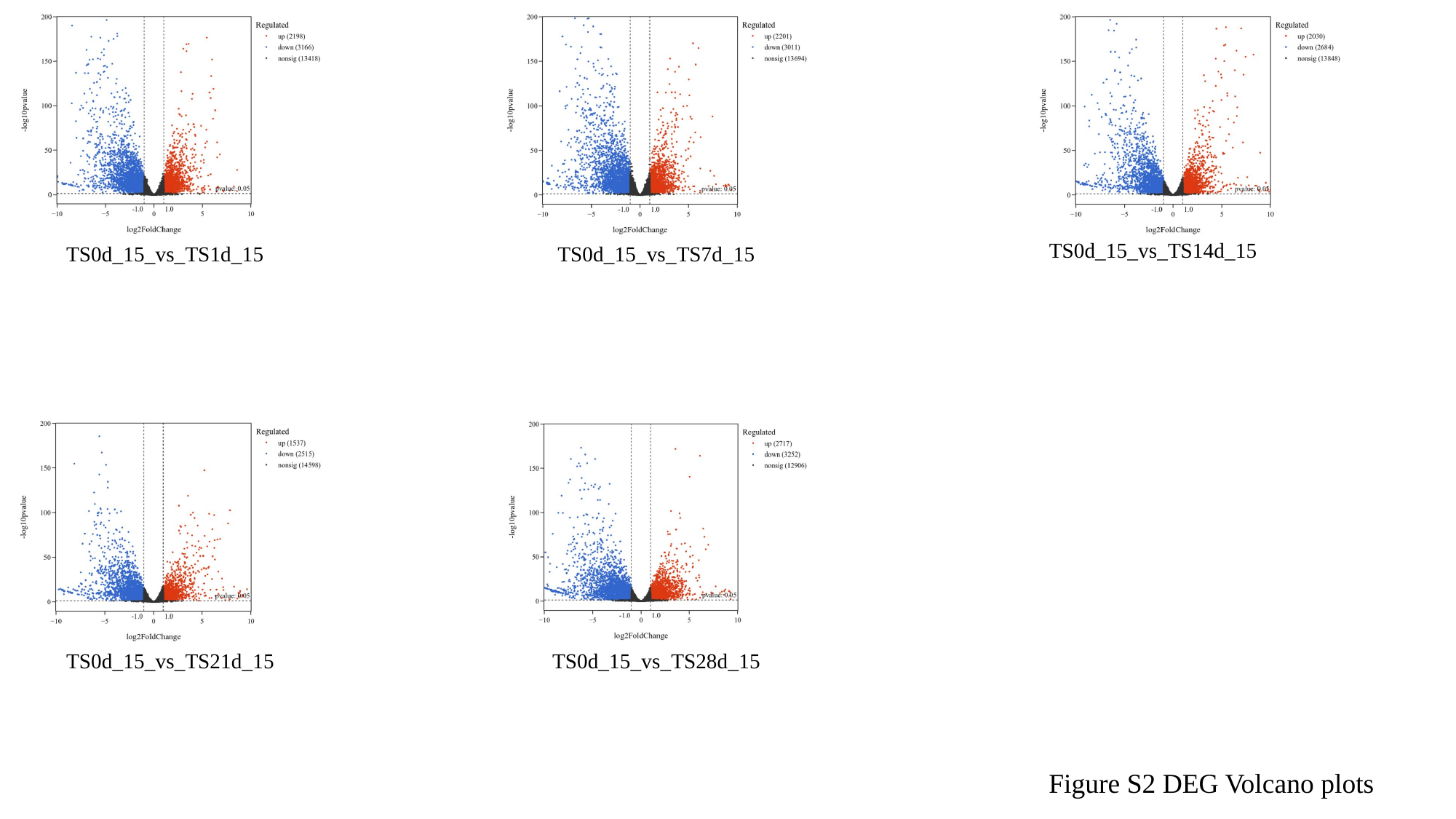

TS0d_15_vs_TS14d_15
TS0d_15_vs_TS7d_15
TS0d_15_vs_TS1d_15
TS0d_15_vs_TS28d_15
TS0d_15_vs_TS21d_15
Figure S2 DEG Volcano plots

## Slide 4
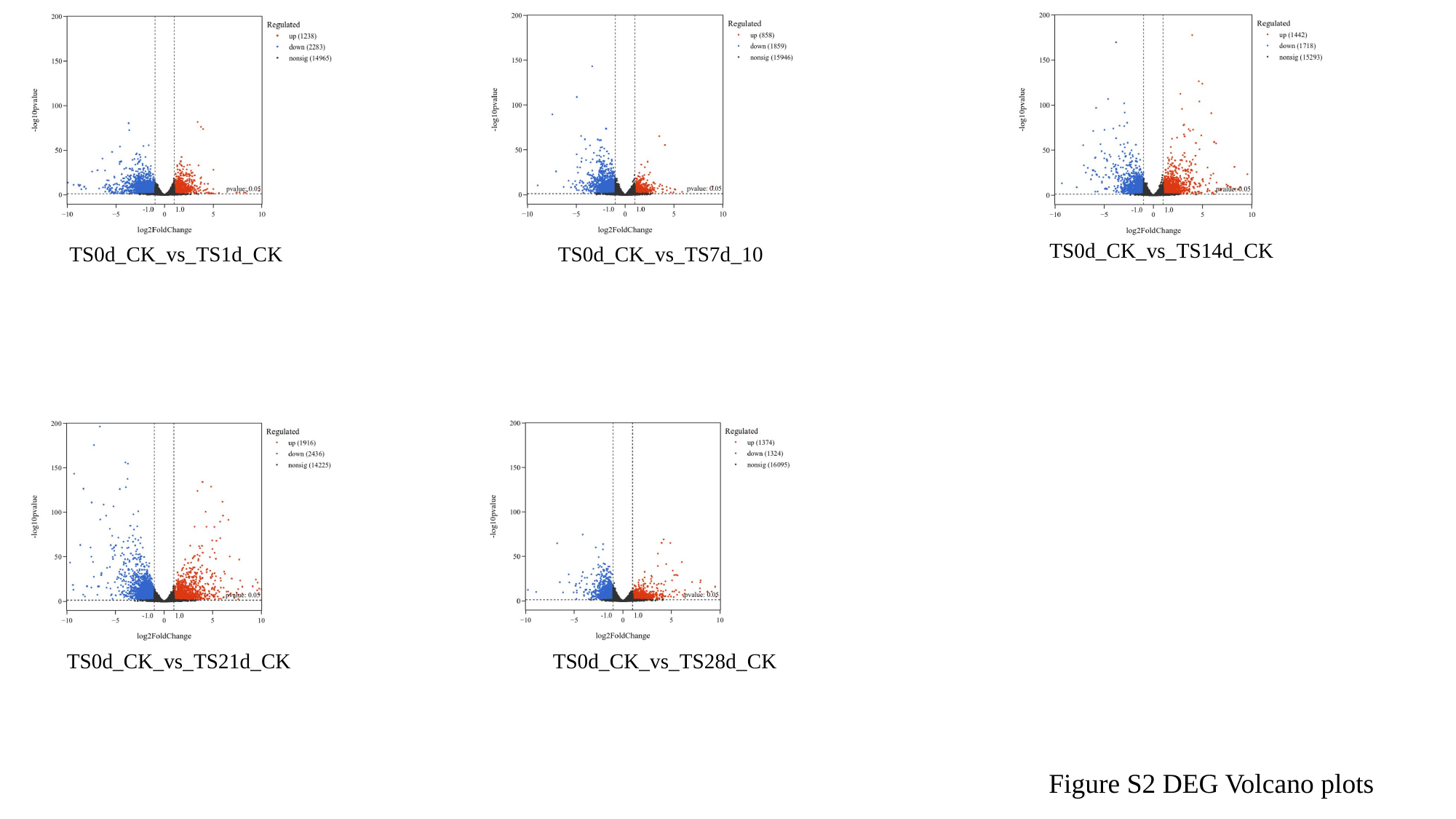

TS0d_CK_vs_TS14d_CK
TS0d_CK_vs_TS7d_10
TS0d_CK_vs_TS1d_CK
TS0d_CK_vs_TS28d_CK
TS0d_CK_vs_TS21d_CK
Figure S2 DEG Volcano plots

## Slide 5
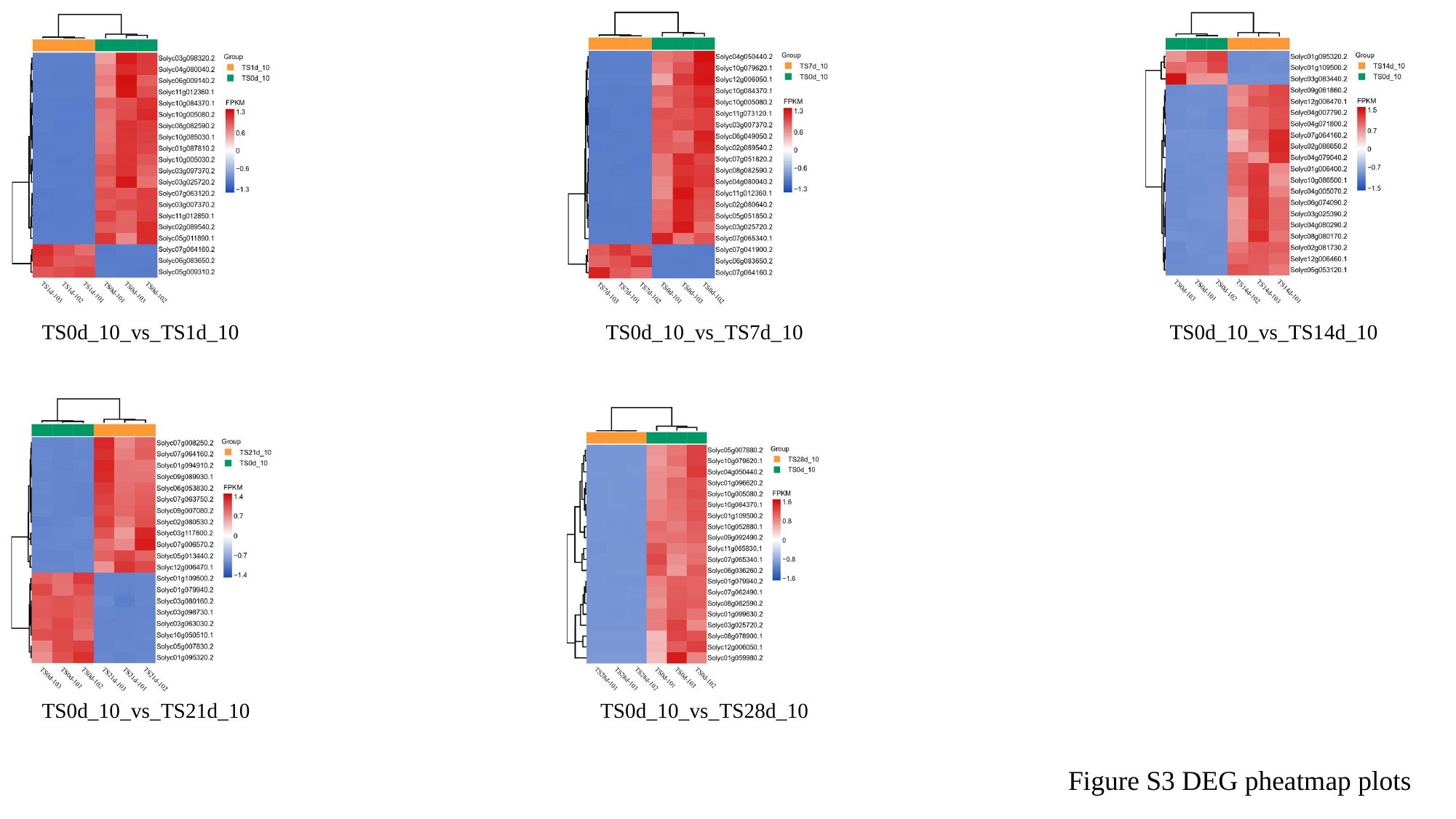

TS0d_10_vs_TS7d_10
TS0d_10_vs_TS14d_10
TS0d_10_vs_TS1d_10
TS0d_10_vs_TS21d_10
TS0d_10_vs_TS28d_10
Figure S3 DEG pheatmap plots

## Slide 6
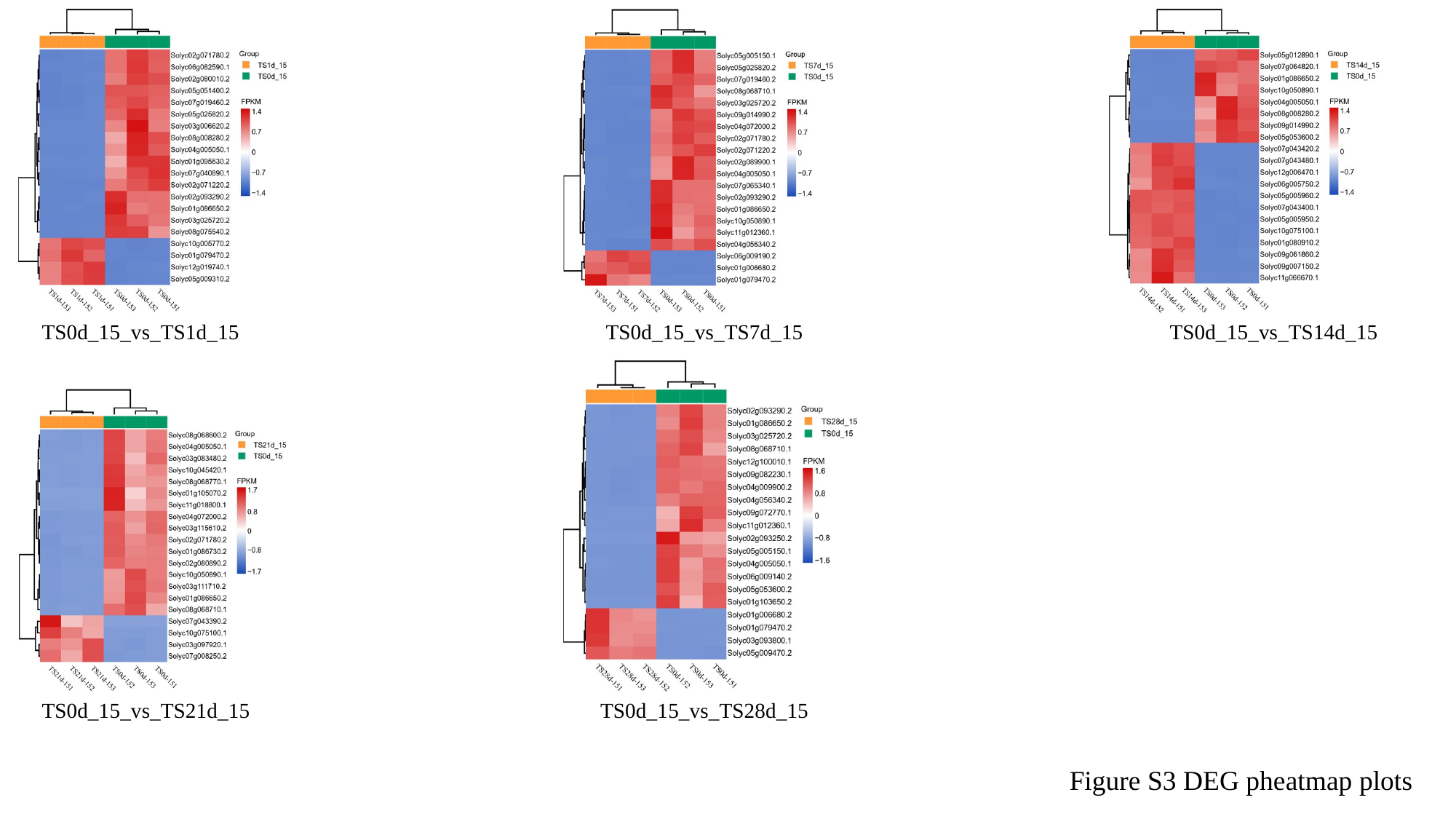

TS0d_15_vs_TS7d_15
TS0d_15_vs_TS14d_15
TS0d_15_vs_TS1d_15
TS0d_15_vs_TS21d_15
TS0d_15_vs_TS28d_15
Figure S3 DEG pheatmap plots

## Slide 7
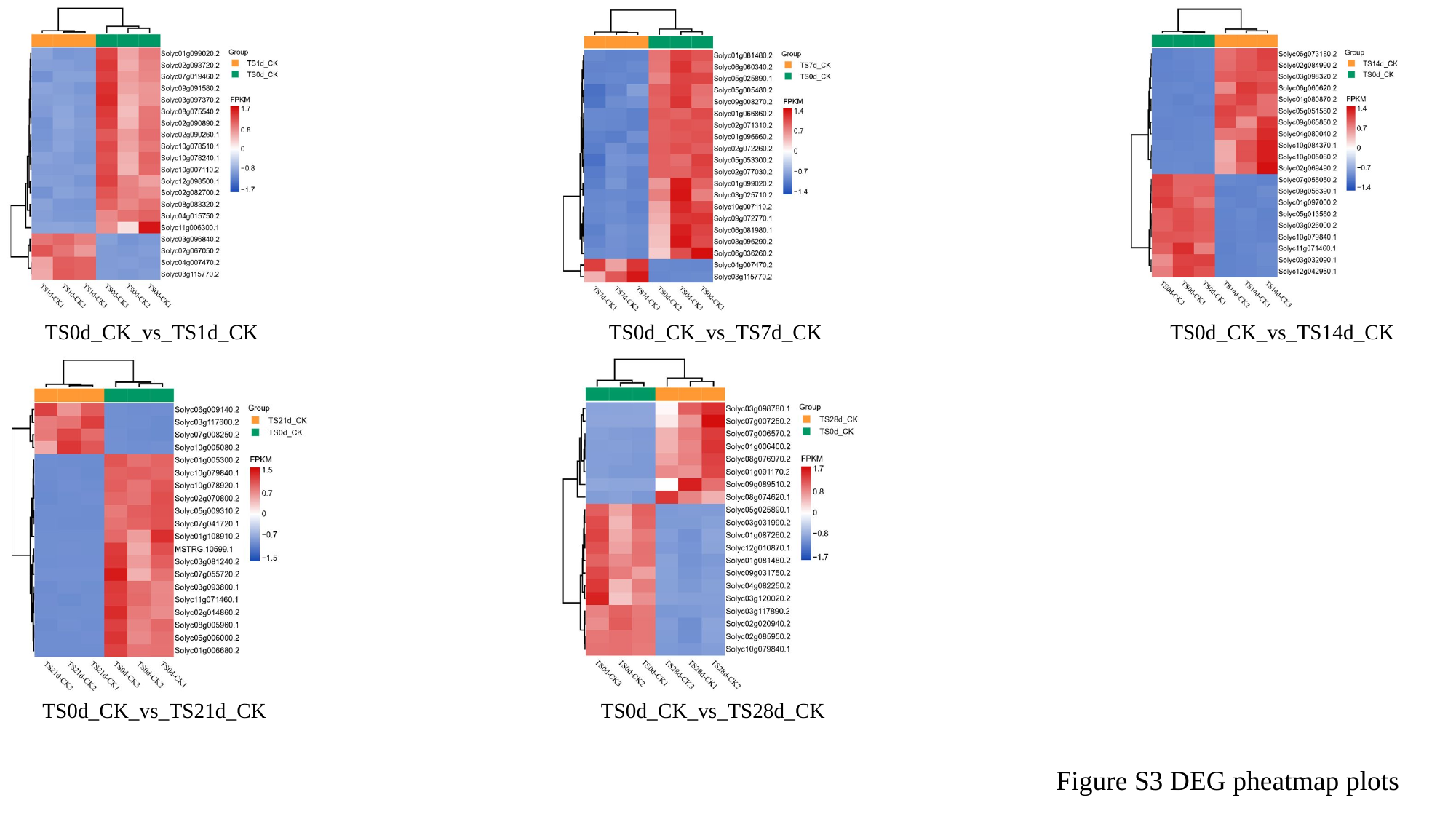

TS0d_CK_vs_TS7d_CK
TS0d_CK_vs_TS14d_CK
TS0d_CK_vs_TS1d_CK
TS0d_CK_vs_TS21d_CK
TS0d_CK_vs_TS28d_CK
Figure S3 DEG pheatmap plots
